# Structural insights into mutagenicity of anticancer nucleoside analog cytarabine during replication by DNA polymerase η

**DOI:** 10.1038/s41598-019-52703-7

**Published:** 2019-11-08

**Authors:** Olga Rechkoblit, Robert E. Johnson, Angeliki Buku, Louise Prakash, Satya Prakash, Aneel K. Aggarwal

**Affiliations:** 1 Department of Pharmacological Sciences, Icahn School of Medicine at Mount Sinai, Box 1677, 1425 Madison Avenue, New York, NY 10029 USA; 2 Department of Biochemistry and Molecular Biology, University of Texas Medical Branch, 301 University Boulevard, Galveston, TX 77755–1061 USA

**Keywords:** Biochemistry, Genetics, Structural biology

## Abstract

Cytarabine (AraC) is the mainstay chemotherapy for acute myeloid leukemia (AML). Whereas initial treatment with AraC is usually successful, most AML patients tend to relapse, and AraC treatment-induced mutagenesis may contribute to the development of chemo-resistant leukemic clones. We show here that whereas the high-fidelity replicative polymerase Polδ is blocked in the replication of AraC, the lower-fidelity translesion DNA synthesis (TLS) polymerase Polη is proficient, inserting both correct and incorrect nucleotides opposite a template AraC base. Furthermore, we present high-resolution crystal structures of human Polη with a template AraC residue positioned opposite correct (G) and incorrect (A) incoming deoxynucleotides. We show that Polη can accommodate local perturbation caused by the AraC via specific hydrogen bonding and maintain a reaction-ready active site alignment for insertion of both correct and incorrect incoming nucleotides. Taken together, the structures provide a novel basis for the ability of Polη to promote AraC induced mutagenesis in relapsed AML patients.

## Introduction

Cytarabine (1-β-D-arabinofuranosylcytosine, AraC) is a nucleoside analog that has remained the backbone of chemotherapy for acute myeloid leukemia (AML) for over 40 years^1–3^. Although initial chemotherapy can be successful in newly diagnosed AML patients, the majority of patients tend to relapse^4,5^. While there are many reasons for relapse, AraC treatment-induced mutagenesis itself can contribute to the development of chemo-resistant leukemic clones^6–10^. Indeed, AraC chemotherapy has a notable effect on the mutational spectrum observed in relapsed AML patients^6–10^.

**Figure 1:**
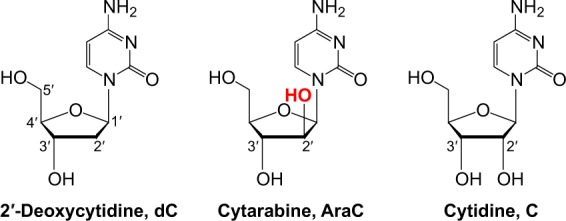
Deoxycytidine, cytarabine, and cytidine nucleosides. Chemical structures of 2′-deoxycytidine (dC, 1-β-D-2′-deoxy-ribofuranosylcytosine), cytarabine (AraC, 1-β-D-arabinofuranosylcytosine), and cytidine (C, 1-β-D-ribofuranosylcytosine). The 2′-OH group in arabinose and ribose sugar moieties points to the opposite directions.

AraC consists of a cytosine base linked to an arabinose sugar (Fig. 1). Thus, it differs from 2′-deoxycytidine (dC) only by the presence of an additional hydroxyl group at the C2′ position of the 2′-deoxyribose. This 2′-OH of the arabinose sugar moiety points in an opposite direction to that of the 2′-OH of the ribose sugar in ribonucleotides (NTPs) (Fig. 1). Upon entering the cell via membrane nucleoside transporters^11^, AraC is phosphorylated by deoxycytidine and pyrimidine kinases to its active form, AraC 5′-triphosphate (AraCTP)^12^.

AraC kills rapidly proliferating cells in the S-phase of the cell cycle primarily by stalling replication forks and generating DNA double stranded breaks^12^. Human high-fidelity replicative DNA polymerases α, δ and ε that synthesize the bulk of genomic DNA^13–15^ can proficiently use AraCTP and insert AraC residue at the 3′ terminus of a growing DNA chain but the subsequent extension reaction is inhibited^16–20^. However, a substantial fraction of AraC-terminated primers do get extended^21–25^; wherein, AraC becomes part of the template strand during the next round of replication and requires lower-fidelity translesion DNA synthesis (TLS) polymerases for its subsequent bypass. Amongst TLS polymerase (Pols), Polη stands out in that human cells deficient in Polη are ~3-fold more sensitive to AraC than wild-type cells^26^.

We show here that high-fidelity replicative Polδ is severely blocked by template AraC. In contrast, TLS polymerase Polη is proficient in the bypass of the AraC on the template strand. While Polη prefers to insert the correct G opposite AraC, misincorporation of mutation-causing non-complementary bases also occurs frequently. To see how Polη can incorporate correct (G) and incorrect (A) deoxynucleotides opposite AraC, we also determined high-resolution crystal structures of human Polη in ternary complexes with AraC and the deoxynucleotide analogs dGMPNPP and dAMPNPP. Surprisingly, despite very different base pair geometries of AraC-dGMPNPP and AraC- dAMPNPP, the Polη active site remains aligned for insertion of correct G as well as mutation-inducing A opposite AraC. Taken together, the structures provide a basis for Polη’s efficient bypass of AraC, as well as a basis for its error-prone synthesis, relevant to an understanding of the mutagenicity of AraC in cells.

## Results

### Biochemical analysis

**Figure 2:**
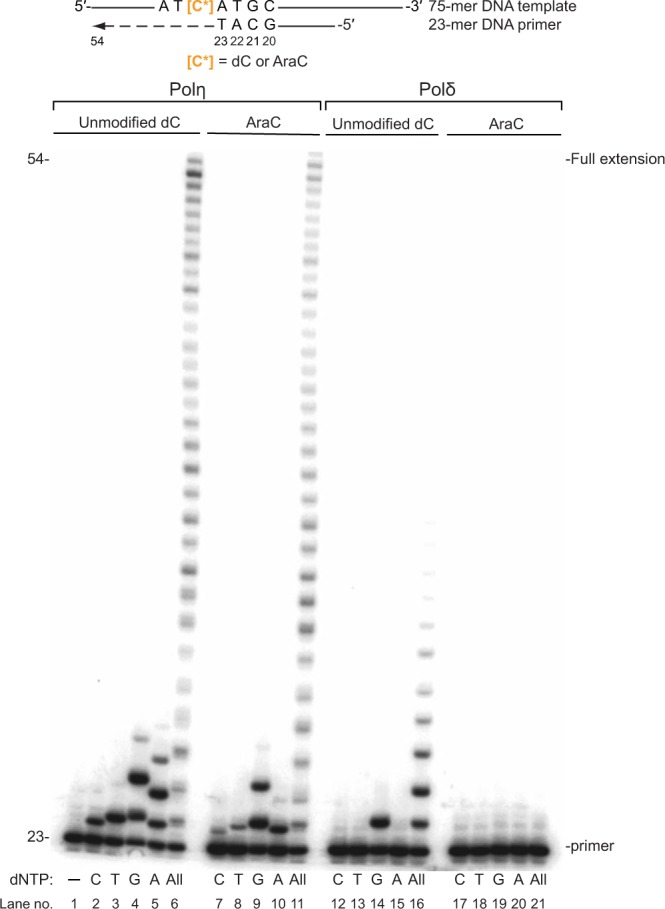
Polη and Polδ-catalyzed primer extension on the unmodified C- and AraC-containing DNA templates. Extension of ^32^P 5′-end-labeled 23-mer primer on the unmodified dC- or AraC-containing 75-mer template. The template–primer (schematics shown on top) creates a “standing start” substrate that allows first nucleotide be incorporated either opposite the unmodified dC or AraC residue and it has a 31-mer 5′–template overhang that permits synthesis up to 54-mer long full extension product. 0.5 nM human full-length Polη or human full-length Polδ holoenzyme (comprised of the PolD1, PolD2, PolD3 and PolD4 subunits) were incubated with 10 nM DNA substrate and 25 μM of either dCTP, dTTP, dGTP, dATP or all four dNTPs for 10 min at 37 °C. Polη-catalyzed reactions are shown in Lanes 1–11, and reactions containing Polδ in Lanes 12–21. Lane 1 has no dNTPs added and shows an unextended primer. The reactions containing only dCTP, dTTP, dGTP or dATP are labeled by C, T, G or A, and the reactions containing all four dNTPs are indicated by All.

We examined the abilities of full-length human TLS Polη and full-length human high-fidelity replicative Polδ holoenzyme (comprised of the PolD1, PolD2, PolD3 and PolD4 subunits) to carry out synthesis on the AraC-containing 75-mer DNA template. This 75-mer DNA template (5′–AGCTACCATG CCTGCCTCAA GAATTCGTAT **X**ATGCCTACA CTGGAGTACC GGAGCATCGT CGTGACTGGG AAAAC–3′, where **X** denotes either dC or AraC) is annealed to the 5′–^32^P labeled 23-mer DNA primer (5′–CTCCGGTACT CCAGTGTAGG CAT–3′). This template-primer complex creates a “standing start” substrate that allows first nucleotide be incorporated either opposite the unmodified dC or AraC residue and has a 31-mer 5′–template overhang that permits synthesis of the 54-mer long full extension reaction product (an 11-mer 3′-template overhang remains single stranded) (Fig. 2). On the unmodified dC template in the presence of all four dNTPs, Polη efficiently carries out DNA synthesis and extends 64% of the primer strands in 10 minutes of the reaction time (Fig. 2, lane 6). In the presence of a single dNTP nucleotide at a time (Fig. 2, lanes 2–5), Polη exhibits its typical error-prone behavior^27,28^. We observe insertion of the correct dGTP into 65% of primer strands, and misincorporation of dATP to 60%, dTTP to 43% and dCTP to 19% of the primer strands. Error-prone nature of Polη also manifests in misincorporation of the same dNTP more than once disregarding the template sequence (Fig. 2, lanes 2–5). On the AraC-containing template in the presence of all four dNTPs, Polη extends about 56% of the primer strand (Fig. 2, lane 11), thus, exhibiting a near equal efficiency as on the unmodified DNA template–primer (Fig. 2, lane 6). In the presence of a single dNTP at a time (Fig. 2, lanes 7–10), Polη incorporates the correct dGTP opposite the AraC slightly slower than opposite the unmodified dC extending ~49% of the primer strands. Furthermore, the misincorporation opposite the AraC is less efficient than opposite the unmodified dC, with dATP, dTTP and dCTP insertion to ~20%, ~6% and ~1% of the primer strands, respectively. Thus, a template AraC residue does not impede Polη-catalyzed DNA synthesis, and dGTP and dATP are the predominant nucleotides inserted.

In contrast, we do not observe any Polδ-catalyzed base incorporation opposite the template AraC residue, similar to that is observed with other high-fidelity polymerases^16,18,29,30^. Thus, on the unmodified template in the presence of all dNTPs, Polδ extends ~44% of the primer strands (Fig. 2, lane 16). The absence of the fully extended products and a low replicative polymerase synthesis rate are in accordance to that observed previously for Polδ holoenzyme in the absence of proliferating cell nuclear antigen (PCNA)^31,32^. We have used a reasonably low Polδ concentration (0.5 nM) to ensure that the primer usage does not exceed 50% and the primer extension is conducted via predominantly single polymerase-DNA binding evens (single-hit conditions)^33^. Such conditions closer approximate a dissociation event Polδ can experience at replication fork. Only an incorporation of the correct dGTP opposite the unmodified dC is detectable on the gel as expected for a high-fidelity enzyme (Fig. 2, lanes 12–15). Significantly, on the AraC template, Polδ exhibited no ability to extend the primer in the presence of all dNTPs (Fig. 2, lane 21) or a single nucleotide (Fig. 2, lanes 17–20). This suggests, that if Polδ would encounter an AraC lesion during replication, it will likely stall and rapidly dissociate from PCNA sliding clamp^34^ giving a way for a lower fidelity TLS Polη to conduct lesion bypass^35^.

### Structure determination

We crystalized the human Polη catalytic core (residues 1 to 432) with a template-primer 12/8-mer (5′–CAT(AraC)ACAGTGCT–3′/5′–AGCACTGT–3′) and non-reactive dGTP analog dGMPNPP. We refined this cognate ternary complex structure with the G opposite the AraC template residue to 2.4 Å resolution and to R_free_ of 21.4% and R_work_ of 17.7%.

**Figure 3:**
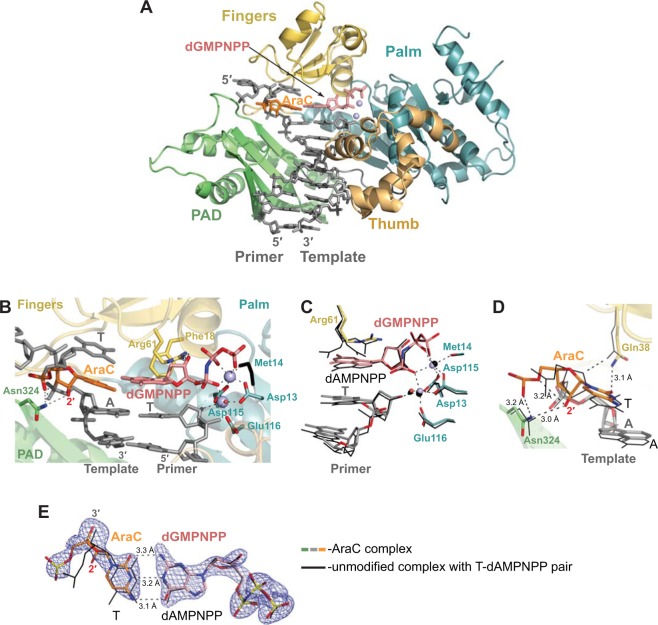
Correct G insertion opposite the AraC ternary Polη complex. (**A**) Overall structure of the complex; the palm, fingers, thumb and PAD domains are shown in cartoon representation in cyan, yellow, light orange, and green, respectively. The DNA template–primer duplex is shown in gray sticks with the template AraC residue in orange. The incoming dGMPNPP residue is in red. The Mg^2+^ ions A and B are represented as light blue spheres. (**B**) A close-up view of the AraC template base in the active site of Polη. Asp13, Asp115, and Glu116 are the catalytic residues. (**C**) Alignment of the 3′-OH primer terminus, incoming nucleotide and active site residues in comparison with unmodified ternary complex with T-dAMPNPP replicating base pair (shown in black lines) (PDB ID: 3MR2)^36^. Both complexes have a T residue at the 3′-end of the primer strand. The structures are superimposed by the palm and fingers domains of the polymerase. (**D**) Alignment of the AraC template residue in comparison with the unmodified template T and interactions of the AraC with Polη. (**E**) A simulated annealing Fo − Fc omit map (contoured at 3.0σ-level at 2.40 Å resolution and colored in blue) showing the clear electron density for the entire AraC residue and its partner base dGMPNPP and comparison with the unmodified base pair. Hydrogen bonds are indicated by dashes and labeled with distances.

**Figure 4:**
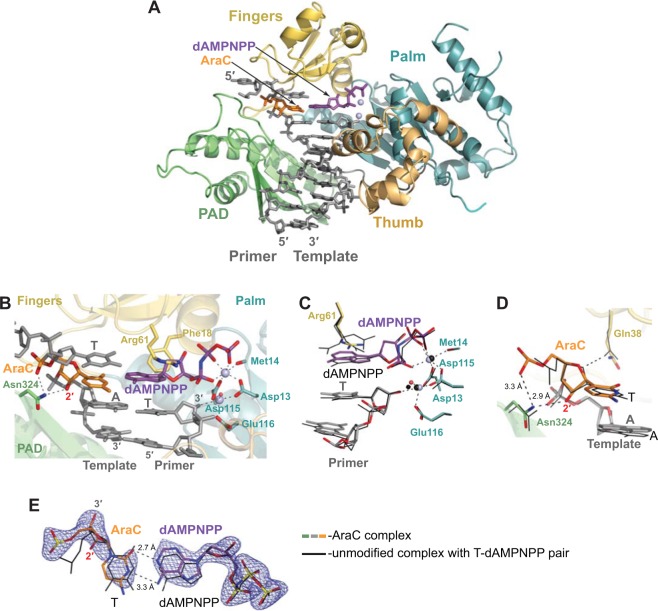
Misinsertion of A opposite the AraC ternary Polη complex. (**A**) Overall structure of the complex; the palm, fingers, thumb and PAD domains are shown in cartoon representation in cyan, yellow, light orange, and green, respectively. The DNA template–primer duplex is shown in gray sticks with the template AraC residue in orange. The incoming dAMPNPP residue is in purple. The Mg^2+^ ions A and B are represented as light blue spheres. (**B**) A close-up view of the AraC template base in the active site of Polη. Asp13, Asp115, and Glu116 are the catalytic residues. (**C**) Alignment of the 3′-OH primer terminus, incoming nucleotide and active site residues in comparison with unmodified ternary complex with T-dAMPNPP replicating base pair (shown in black lines) (PDB ID: 3MR2)^36^. Both complexes have a T residue at the 3′-end of the primer strand. The structures are superimposed by the palm and fingers domains of the polymerase. (**D**) Alignment of the AraC template residue in comparison with the unmodified template T and interactions of the AraC with Polη. (**E**) A simulated annealing Fo − Fc omit map (contoured at 3.0σ-level at 2.10 Å resolution and colored in blue) showing the clear electron density for the entire AraC residue and its partner base dAMPNPP and comparison with the unmodified base pair. Hydrogen bonds are indicated by dashes and labeled with distances.

To visualize the misinsertion of an A opposite the template AraC we first tried, unsuccessfully, to grow crystals with the same template-primer DNA as described above for the cognate complex while replacing dGMPNPP by dAMPNPP. We chose to facilitate crystallization by modifying the DNA duplex within the polymerase-unbound end and introduced a mismatch to loosen the end-to-end DNA packing interactions. We then succeeded with the template-primer (5′–CAT(AraC)ACAGTGCG–3′/5′-AGCACTGT-3′). We refined the mismatched complex structure to 2.1 Å resolution and to R_free_ of 23.8% and R_work_ of 19.6%. Both structures provide atomic details on the conformation of AraC residue and its interactions with the polymerase (Figs 3A–E and 4A–E). The crystal data, data collection statistics, and refinement statistics for both complexes are summarized in Table 1.

**Table 1:** X-ray data collection and refinement statistics.

	Correct G insertion opposite AraC ternary complex	Misinsertion of A opposite AraC ternary complex
Data collection		
Space group	P6_1_	P6_1_
Cell dimensions:		
*a, b, c* (Å)	98.2 98.2 81.3	99.0 99.0 81.8
α, β, γ (°)	90.0, 90.0, 120.0	90.0, 90.0, 120.0
Resolution range (Å)^a^	45.0–2.40 (2.44-2.40)	85.7-2.09 (2.09-2.15)
*R*_merge_ (%)	11.0 (88.4)	13.3 (17.8)
*I*/σ*I*	29.9 (4.0)	11.6 (1.0)
Completeness (%)	100.0 (100)	100 (100)
Redundancy	19.1 (19.5)	6.8 (6.9)
CC_1/2_ (%)	100.0(87.6)	99.7 (34.8)
**Refinement**		
Resolution range (Å)	42.5-2.40	59.2-2.09
No. reflections	17,623	27,040
*R*_work_/*R*_free_	17.4/21.6	19.6/23.8
No. atoms		
Protein	3,332	3,374
DNA	346	388
Ligand (dNMPNPP)	31	30
Ligand (other)	6	12
Ion (Mg^2+^)	2	2
Water	123	214
*B*-factors		
Protein	37.3	45.4
DNA	42.5	46.8
Ligand (dNMPNPP)	37.9	39.7
Ligand (other)	28.3	41.2
Ion (Mg^2+^)	37.2	39.6
Water	33.3	44.4
R.m.s. deviations		
Bond length (Å)	0.011	0.011
Bond angles (°)	0.83	0.69

^a^Values in parentheses are for highest-resolution shell.

### Overall arrangement

In both AraC-containing ternary complexes (Figs 3A and 4A), Polη encircles the template–primer with its palm (residues 1–13 and 90–238), fingers (residues 17–87), thumb (residues 241–301) domains as well as the PAD (polymerase associated domain; residues 319–432). The palm domain carries the catalytic residues Asp13, Asp115, and Glu116, while the fingers domain lies above the templating AraC base (Figs 3B and 4B). The thumb and the PAD grasp the template–primer DNA duplex at opposite sides, from the minor and major groove surfaces, respectively (Figs 3A and 4A). As in the ternary structures of Polη with unmodified DNA templates^36^, the sugar moieties of the incoming dGMPNPP and dAMPNPP nucleotides are packed against the aromatic ring of Phe 18, which acts as a “streric gate” for the exclusion of ribonucleotides. The triphosphate moieties of dGMPNPP and dAMPNPP are interlaced between the fingers and palm domain and assume the same conformation as in the unmodified Polη complexes (Figs 3B,C and 4B,C).

### Insertion of correct G opposite AraC

The AraC base pairs with the G base of incoming dGMPNPP with the expectant Watson-Crick (W-C) geometry (Fig. 3E**)**. However, the C1′- C1′ distance across the base pair is ~10.71 Ả, as compared to 10.5 Ả for an ideal W-C base pair. Also, the AraC base is tilted by ~30°, which negatively impacts its stacking with the adjacent bases on the template strand (Fig. 3D**)**. The AraC sugar assumes the C1′-*exo* conformation rather than the C2′-*endo* conformation observed in Polη structures with unmodified DNA. C1′-*exo* is one of the preferred sugar conformations for arabinonucleosides^37^, and it appears to be further stabilized in the Polη active by a hydrogen bond between the “extra” 2′-OH on the AraC sugar and the main chain amide of Asn324 of the polymerase (Fig. 3D). Together, this lends to further local adjustments in the AraC sugar and the active site residues (when compared to the unmodified structures), including an ~1.0 Ả shift in the O4′ atom of the AraC sugar and a relocation of the AraC phosphate group by ~1.4 Ả, with the latter now in a position to make a hydrogen bonds with amide group of Asn324 and giving rise to an unusual intramolecular hydrogen bond between the C2′-OH and the O5′ atoms of AraC (Fig. 3D**)**. Also, in contrast to the unmodified structures, the side chain of Gln38 forms a hydrogen bond with the *O*^2^ atom of the AraC base instead of the O4′ atom the sugar, and Arg61 (unique to Polη) adopts a single rotameric conformation to stabilize the binding of the incoming nucleotide rather than multiple conformations.

In spite of these local rearrangements, the Polη active site is well adapted for the incorporation of the incoming nucleotide opposite template AraC. Analogous to the unmodified structure, the catalytic Mg_A_^2+^ ion is coordinated by the 3′-OH of the primer terminus, the α-phosphate group oxygen atom of dGMPNPP, the carboxylates of Asp115 (2.14 Ả), Glu116 (1.89 Ả), and Asp13 (2.09 Ả), and a water molecule (Fig. 3C). Mg_B_^2+^ is ligated by the dGMPNPP β- and γ-phosphates, the carboxylates of Asp13 (1.95 Ả) and Asp115 (2.18 Ả) and by the backbone carboxyl oxygen of Met14 (2.34 Ả). Importantly, the 3′-OH of the primer terminus is at the reaction-ready distance of 3.41 Ả from the α-P atom of dGMPNPP. Thus, despite local adjustments in how AraC is accommodated in the Polη active site, the polymerase is well poised for the incorporation of G opposite template AraC.

### Insertion of mismatched A opposite AraC

**Figure 5:**
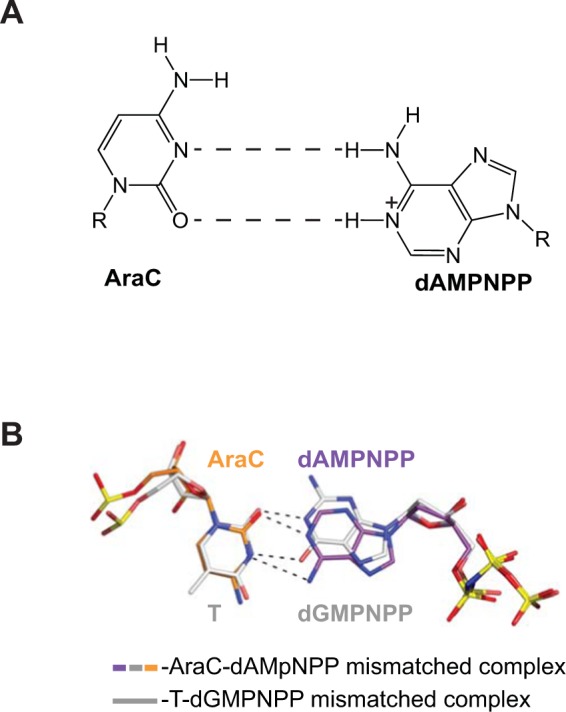
AraC wobble mispair with A and comparison with the T:G. (**A**) A schematics of the wobble AraC:A base pair observed in the misinsertion AraC Polη complex. (**B**) Wobble AraC:A and T:G base pairs in the active site of Polη. The T:G containing Polη complex (PDB ID: 4J9K)^39^ has been superimposed to the AraC:A complex by the palm and fingers domains of the polymerase. The AraC:A and T:G base pairs are shown in sticks and colored by atom. The carbon atoms are colored differently: orange in AraC, purple in dAMPNPP, white in T and dGMPNPP.

AraC forms a wobble base pair with the A base of the incoming dAMPNPP (Fig. 4E). From the observed geometry, the adenine base of the dAMPNPP is likely protonated at the N1 position, resulting in a 2.7 Å hydrogen bond with the acceptor *O*^2^ atom of the AraC base (Fig. 5A). A second hydrogen bond forms between the N3 acceptor group of the AraC and the donor atoms *N*^6^ (3.3 Å) of dAMPNPP. The overall geometry is similar that of a C:A mismatch in a free DNA duplex^38^, including ~0.65 Ả shifts in the AraC/C and dAMPNPP/A bases towards the major and minor grooves of the DNA, respectively, when compared to a W-C base pair. Because of the wobble base pair, the AraC residue is too far away to make contacts with the side-chain of Gln38 and the base is untilted and maintains stacking with the adjacent DNA bases (Fig. 4D). The C1′- C1′ distance across the base pair is 10.33 Ả, typical for wobble base pair.

Despite these local adjustments to accommodate a mismatched AraC-A base pair, the Polη active site is relatively unperturbed and the 3′-OH of the primer terminus is at the reaction-ready 3.55 Ả distance from the α-P atom of dAMPNPP (Fig. 4B,C). Also, as in the cognate complex, the AraC sugar pucker is C1′-*exo*, the Asn324 side-chain forms a direct hydrogen bond with the 2′-OH of the sugar, and the phosphate group of the AraC is positioned to make contact with Asn324 (though, too far away for an intramolecular hydrogen bond between the C2′-OH and the O5′ atoms) (Fig. 4D).

## Discussion

AraC has remained the mainstay chemotherapy for AML for over 40 years^1–3^. The incorporation of AraC into the genome results in blockage of the high-fidelity polymerases at the sites of incorporation and necessitates the participation of TLS Pols in its subsequent bypass. We show here that human Polη can efficiently bypass AraC embedded in a DNA template strand by incorporating cognate G as well as noncognate deoxynucleotides (predominantly an A) opposite the lesion. We provide a structural basis for this ability of human Polη to insert both a correct (G) and an incorrect (A) deoxynucleotide opposite AraC. Surprisingly, despite very different base pair geometries, W-C for AraC-G and wobble for AraC-A, the plasticity of the Polη active site is such that it permits the catalytic residues to remain aligned for the insertion of correct G as well as mutation-inducing A opposite AraC.

Although chemotherapy with AraC is very successful for newly diagnosed AML patients, the majority of patients tend to relapse. Thus, treatment of AML with AraC is in many ways a double-edged sword. Studies of AraC exposure in human TK6 cells have established AraC as a mutagen^7^, and relapsed patients have been shown to carry a higher AraC-induced mutation burden^7–10^. Notably, AraC is suggested to be a base substitution mutagen because DNA mismatch repair (MMR) deficient cells had higher mutation frequencies than their MMR-proficient counterparts^7^. Based on our studies, part of this mutation burden likely arises from error-prone replication of AraC by human Polη. Polη is particularly effective in inserting A opposite AraC, though mutagenic C and T also get inserted. The insertion of A does not perturb the active site sufficiently to preclude the catalytic reaction. The putative primer 3′OH remains well positioned for a nucleophilic attack on the α-P atom of the mismatched nucleotide. This was of surprise because a T:G wobble base pair by contrast, which is often formed by Polη to promote somatic hypermutation^39^ (Fig. 5B) leads to a primer 3′-OH that is predominantly in a nonproductive conformation^39^.

Taken together, Polη emerges from our studies as capable of promoting AraC induced mutations during chemotherapy. It has also been shown that AML relapse can originate from leukemia clones that exist prior to chemotherapy and have greater transcriptional plasticity^40^. It would be interesting to evaluate if these highly adaptive clones upregulate expression of Polη to promote survival during the second round of treatment.

## Methods

### Preparation of proteins for biochemical studies

To express full-length human Polη in yeast, the PolH cDNA in clone GS27149^41^ was amplified by PCR and cloned in frame with the Glutathione S-transferase gene under control of a galactose inducible phosphoglycerate kinase (PGK) promoter in plasmid pBJ842^42^, generating plasmid pR30.186. The integrity of PCR generated regions was confirmed by sequencing. Yeast strain YRP654 was transformed with pR30.186 and human Polη protein was expressed and purified as described^43^. To produce human full-length four-subunit Polδ holoenzyme, the p125, p50, p66 and p12 subunits of Polδ were co-expressed in yeast from 3 plasmids. The PolD1 cDNA encoding the p125 catalytic subunit was amplified by PCR from a baculovirus expression vector^44^ (gift from Dr. Ellen Fanning). The PolD2, PolD3 and PolD4 cDNAs encoding the p50, p66 and p12 subunits, respectively, were each amplified by PCR from baculovirus expression vectors obtained from Dr. Jerard Hurwitz. Each cDNA was confirmed by sequencing. The PolD1 cDNA was cloned in frame with a Flag-metal affinity tag in plasmid pPM1257, which harbors the yeast *leu2d* gene, generating pBJ1604 plasmid. The PolD2 cDNA was cloned in frame with Glutathione S-transferase gene in plasmid pBJ842 and the PolD3 gene was expressed natively by cloning in plasmid pBJ1179, which carries the *Trp1* gene. The GST-PolD2 expression cassette was then subcloned into the PolD3 expression plasmid generating the dual GST-PolD2/PolD3 Trp1 yeast expression plasmid, pBJ1599. PolD4 was expressed natively by cloning the cDNA into the *Ura3* containing plasmid pPM271, generating pBJ1601 plasmid. Yeast strain YRP654 was co-transformed with plasmids pBJ1604, pBJ1599 and pBJ1601 and colonies were selected for on synthetic yeast media lacking leucine, uracil and tryptophan. Protein expression was carried out as described^42^. The hPolδ holoenzyme was purified using a standard protocol utilizing glutathione Sepharoase (GE biotech) and anti-Flag M2 Agarose (Sigma) affinity purification steps^42^. All tags were removed from the respective fusion proteins by PreScission protease.

### Primer extension

DNA substrates consisted of a 75-mer template DNA template (5′–AGCTACCATG CCTGCCTCAA GAATTCGTAT **X**ATGCCTACA CTGGAGTACC GGAGCATCGT CGTGACTGGG AAAAC–3′, where **X** denotes either dC or AraC) and a 5′–^32^P labeled 23-mer primer (5′–CTCCGGTACT CCAGTGTAGG CAT–3′). Thus, this template–primer creates a “standing start” substrate that allows the first nucleotide to be incorporated either opposite the unmodified dC or AraC and it has a 31-mer 5′–template overhang that permits synthesis of a 54-mer long full extension reaction product (an 11-mer 3′–template overhang remains single stranded). The 75-mer AraC-modified template was purchased from Midland Certified Reagent Company. DNA 5′– ^32^P-radiolabeled primer was mixed with the unmodified dC- or AraC-containing template in 1:1.5 molar ratio and annealed by heating the solution to 95 °C and allowing it to cool to room temperature for several hours. The DNA polymerase assay was performed as described previously^42^. Reactions (5 μL final volume) contained 25 mM tris-HCl pH 7.5, 0.1 mg/ml BSA, 10% glycerol, 1 mM DTT, 5 mM MgCl_2_, 10 nM DNA substrate, and 25 μM of either dATP, dGTP, dTTP, or dCTP or all four dNTPs combined. Human Polη and Polδ holoenzyme were each assayed at a final concentration of 0.5 nM. Reactions were initiated by the addition of 1 μL of 2.5 nM DNA polymerase solution in 5x reaction buffer (125 mM Tris-HCl pH 7.5, 0.5 mg/ml BSA, 5 mM DTT) to 4 μl of DNA substrate/dNTP/Mg^2+^/glycerol mixture and carried out for 10 minutes at 37 °C before terminating with 6 volumes of loading buffer (95% formamide, 0.06% xylene cyanol and 0.06% bromophenol blue). Reaction products were separated on 15% polyacrylamide gel prepared with Tris-Boric Acid-EDTA (TBE) buffer and containing 8 M urea. Gels were dried and products were visualized by phosphorimaging on a Typhoon FLA7000 (GE biotech).

### Preparation of protein for crystallization

An N-terminal His_6_ tagged catalytic core of human Polη (residues 1–432) with a C406M mutation was overexpressed in *Escherichia coli* and purified as previously described^36,45^. Briefly, the His_6_ tag was removed by overnight incubation with PreScission protease, and the protein was purified by ion-exchange (MonoS) chromatography followed by size-exclusion (Superdex 75). The protein was concentrated to ~1.3 mg/ml in 25 mM tris (pH 8.0), 250 mM NaCl, and 2 mM tris(2-carboxyethyl) phosphate (TCEP) and stored in aliquots at −80 °C.

### Crystallization

The crystals of the ternary complex with the correct incoming guanine opposite the template AraC residue were obtained by incubating the human Polη catalytic core with a DNA template–primer (5′–CAT(AraC)ACAGTGCT–3′/5′-AGCACTGT-3′) (TriLink Biotechnologies Inc. and Glen Research, Inc, respectively) in the presence of non-hydrolysable dGTP analog dGMPNPP (2′-deoxyguanosine-5′[(α,β)-imido]triphosphate, Jena Bioscience) by the hanging drop method against a reservoir solution containing 0.1 M MES pH 6.0 buffer and 10–14% PEG1500. The crystal growth and harvesting were performed as described in our study of AraC incorporation into the primer strand by Polη^46^. Briefly, the template-primer DNAs were annealed by heating for 5 min at 90 °C and slowly cooled to 4 °C and then mixed with Polη in a 1.2:1 molar ratio to ~0.02 mM concentration of the complex in 25 mM tris (pH 8.0), 125 mM NaCl and 1 mM TCEP. The complex was incubated on ice for 20 minutes and then concentrated with Amicon Ultra centrifugal filter (cut-off 3KDa) to a final complex concentration of ~0.105 mM at 4 °C. dGMpNPP and MgCl_2_ were then added to the complex to 2 mM and 4 mM concentrations, respectively. The resulting complex was either used for crystallization immediately or stored in aliquots at −80 °C. In both cases, the complex was centrifuged at 8,000 rpm for 2 min at 4 °C prior to crystallization. The hanging crystallization drop was formed by mixing 1 μL of the complex with 1 μL of the reservoir solution on a siliconized coverslip and the crystals were grown at 20 °C. To produce larger diffraction-quality crystals, a round of microseeding with Seed Bead kit (Hampton Research) was performed. The crystals were scooped out of crystallization drops in CryoLoops (Hampton Research) and cryoprotected in the reservoir solution increased to 24% PEG1500 followed by additional stepwise supplementation with 5%, 10% and 20% glycerol. The cryoprotected crystals were flash frozen in liquid nitrogen for X-ray data collection.

To produce ternary complex crystals visualizing the insertion of an A base opposite the template AraC, we used the template-primer (5′–CAT(AraC)ACAGTGCG–3′/5′-TGCACTGT-3′) DNA and dAMPNPP (2′-Deoxyadenosine-5′-[(α,β)-imido]triphosphate, Jena Bioscience). The crystallization drops were set up, and crystal grown and harvested as described above.

### Structure determination and refinement

The X-ray diffraction data were collected at the NSLS X25 beam line at the Brookhaven National Laboratory and at the 24-ID NE-CAT beamline at Advanced Photon Source in Chicago. The data from the NSLS X25 beam line were processed and scaled using the HKL2000 suite^47^ and the data from the 24-ID NE-CAT beamline were processed by RAPD pipeline (http://necat.chem.cornell.edu/). We solved the structure of the Polη cognate ternary complex with the correct G opposite the template AraC base by the molecular replacement method (Phaser)^48^ in the CCP4 program package^49^ using the Polη extension ternary complex structure with AraC residue at the 3′-end of the primer strand (PDB ID: 6D0Z) as a search model^46^. The model building, including substitution of the DNA sequence, was finished manually in Coot^50^ based on the electron density maps calculated in PHENIX Refine^51^. The final model was refined in PHENIX Refine to 2.4 Å resolution^51^ and belongs to P6_1_ space group with unit cell dimensions of a = b = 98.2 Å, c = 81.3 Å, α = β = 90°, and γ = 120.0°. The structure is refined to R_free_ of 21.4% and R_work_ of 17.7% and consists of one Polη molecule (residues 1 to 432), one DNA template (residues 2 to 12), one DNA primer (residues 1 to 8), one dGMNPP, two Mg^2+^ ions and a total of 122 solvent molecules. The placement and conformation of the AraC residue was verified using simulated annealing omit maps calculated in PHENIX^51^ with the AraC omitted from the model before heating to 2,000 K and then slowly cooling.

The crystals with the incoming A opposite the template AraC diffracted to 2.09 Å resolution and belong to P6_1_ space group with unit cell dimensions of a = b = 99.0 Å, c = 81.8 Å, α = β = 90°, and γ = 120.0°. We solved the structure by MR using the ternary complex with the correct incoming nucleotide (described above) as a search model. The structure is refined to R_free_ of 23.8% and R_work_ of 19.6% and consists of one Polη molecule (residues 1 to 432), one DNA template (residues 2 to 12), one DNA primer (residues 1 to 8), one dGPMNPP, two Mg^2+^ ions and a total of 234 solvent molecules.

The crystal data, together with the data collection and refinement statistics, are summarized in Table 1.

## Data Availability

Atomic coordinates and structure factors have been deposited in the Protein Data Bank under accession codes 6PZ3 and 6Q02 for the correct G and mutation inducing A insertion AraC ternary complexes, respectively. Other data are available from the corresponding author upon reasonable request.
